# Different techniques of adenoidectomy and its impact on middle ear pressure: a randomized controlled study

**DOI:** 10.1007/s00405-023-08188-2

**Published:** 2023-11-06

**Authors:** Ahmed Mohamed Seleim, Ahmed Nabil Elsamnody, Ahmed Fawzy Amer

**Affiliations:** Department of Otorhinolaryngology, Al-Azhar University Hospitals, Al-Azhar University, Cairo, Egypt

**Keywords:** Adenoidectomy, Coblation, Microdebrider, Tympanometry

## Abstract

**Objectives:**

The aim of this work is to compare between different techniques of adenoidectomy: endoscopic microdebrider-assisted, coblation and conventional adenoidectomy and its effect on middle ear pressure.

**Background:**

Adenoidectomy, either alone or with tonsillectomy, is considered among the most performed procedures in pediatric otorhinolaryngology. This procedure usually related to the Eustachian tube function and middle ear status. Eustachian tube dysfunction is mainly caused by mechanical obstruction of the tubal orifice, insufficient swallowing and inflammation in the nasopharyngeal mucosa.

**Methods:**

This prospective randomized study was conducted on 90 patients with symptomatic adenoid hypertrophy confirmed by nasopharyngeal X-ray and endoscopic grading preoperatively. Patients were admitted at Otorhinolaryngology department of our institute during the period from January 2022 to January 2023. They were divided into three groups that were operated either by conventional (Group I), endoscopic microdebrider (Group II), or coblation technique (Group III). Each group was assessed through the audiometric parameters plus postoperative bleeding, and VAS results for pain score and postoperative endoscopic grading for adenoid recurrence.

**Results:**

Mean age in group A was 9.03 years and in group B was 8.99 years and was 8.99 years in group C with insignificant differences between three groups. There is significant improvement of tympanographic results comparing all groups of the patients at 6 months postoperatively. There is significant relation between the mean VAS comparing preoperative and postoperative results.

**Conclusion:**

There are better results in tympanographic data at conventional adenoidectomy versus other techniques. However, there are also better postoperative results after either coblation or endoscopic microdebrider adenoidectomy over the conventional technique.

## Background

The adenoid also known as nasopharyngeal tonsils is a lymphatic tissue in the nasopharynx. It is situated in the midline on the roof and posterior wall of the nasopharynx. The adenoids are midline structures situated on the roof and posterior wall of the nasopharynx [1].

Adenoid hypertrophy is one of the common causes of upper airway obstruction in children. Sleep-related breathing disorder due to adenoid is often seen in children aged 4–5 years, because at this age, the adenoid tissue and palatine tonsils have reached their largest size, untreated adenoid hypertrophy can lead to serious conditions such as maxillofacial anomalies, pulmonary hypertension and cor-pulmonale [2].

Adenoidectomy, either alone or with tonsillectomy, is considered among the most performed procedures in pediatric otorhinolaryngology. Classical adenoidectomy is referred to as the curette adenoidectomy or conventional adenoidectomy, which utilizes a curette for adenoids removal [1].

Afterwards, many alternative techniques have been suggested, including suction electrocautery ablation, laser adenoidectomy, and microdebrider-assisted adenoidectomy. Curettage technique that is widely used currently, remains quite popular. However, it has various complications, such as incomplete removal, trauma to underlying tissues and hemorrhage [3].

Endoscopes began to be utilized in adenoidectomy operations to ensure full removal of the adenoid bulk, achieve better hemostatic control with enhanced visualization and prevent possible damage [4].

Eustachian tube dysfunction is most commonly caused by mechanical obstruction of the tubal orifice, insufficient swallowing and inflammation in the nasopharyngeal mucosa [5]. Eustachian dysfunction can develop due to surgical trauma, edema in surrounding tissues and clots in early period following adenoidectomy surgery performed with curettage technique. Middle ear pressure is affected in early period after adenoidectomy in adenoid hypertrophy patients with normal middle ear pressure. Our study aims to analyze the changes in middle ear pressure in early period after adenoidectomy in children with adenoid hypertrophy [6].

Our aim of this work is to compare between two different techniques of adenoidectomy: endoscopic microdebrider-assisted, coblation, and conventional adenoidectomy and its effect on middle ear pressure.

## Methods

This prospective randomized study was conducted on 90 patients with symptomatic adenoid hypertrophy such confirmed by nasopharyngeal X-ray and endoscopic grading preoperatively. Patients were selected from general population including patients reporting to Otorhinolaryngology outpatient clinics of Al-Azhar University hospitals during the period from January 2022 to January 2023. It is approved by the ethical committee of the University with ethical No. (412/ENT/2022).

Inclusion criteria were only children aged from 3 to 12 years old with symptomatic adenoid hypertrophy, children with sleep disordered breathing due to adenoid hypertrophy, tonsillectomy candidates that were assessed for the presence of asymptomatic adenoid hypertrophy, and these patients had no history of attack of otitis media in the last 6 months.

Exclusion criteria were patients having significant nasal obstruction due to other causes such as: (allergic rhinitis, septal deviation, hypertrophied inferior turbinates or sinonasal polyps), patients with congenital nasal or maxillofacial anomalies such as: cleft lip and palate, choanal atresia, retrognathia or macrognathia, or patients with coagulation disorder, plus patients with past history of myringotomy, grommet tube, recurrent otitis media with effusion.

Patients were subjected to the following: (1) detailed history taking and clinical examination. (2) radiological evaluation of the adenoid by X-ray lateral view on the nasopharynx. (3) endoscopic grading of adenoid size using flexible endoscopy (4) tympanometry for all patients.

Preoperatively, before general anesthesia fiberoptic adenoid endoscopy was done with a 2.7 mm fiberoptic endoscope (Karl Storz, Germany). The aim of this examination is to detect the adenoid size grading as four grades as following: when only upper part of nasopharynx (free choanae) occupied by adenoid mass was classified as grade 1 (≤ 25%). Adenoid tissue occupied equal or less than 50% of nasopharynx with sufficient choanal opening and sufficient tubal visualization was classified as grade 2. If adenoid tissue occupies about 75% of the nasopharynx with partial involvement of the tube ostium and considerable obstruction of choanal openings was classified as grade 3. If adenoid tissue reaches the lower choanal border without allowing visualization of the tube ostium (> 75%) was classified as grade 4 [7].

All patients underwent polysomnography before adenoidectomy to assess the presence of sleep disorder; then under general anesthesia all patients were operated either as a single procedure or along with other procedures like tonsillectomy. The patients were tabulated on computer and depending on double blind they were divided randomly into three groups: Group I of 30 patients had conventional adenoidectomy, Group II of 30 patients had endoscopic microdebrider-assisted adenoidectomy, and Group III of 30 patients had coblation adenoidectomy.

Intraoperative endoscopic nasal examination after adenoidectomy was done to evaluate the presence of adenoid remanent in both groups and to control bleeding. Patients were recorded for operative time, operative and post-operative complications. Pediatric flexible nasopharyngoscopy was done for all patients 6 months postoperatively for re-assessment of the nose and nasopharynx. Informed written consent was obtained from all patients.

Conventional adenoidectomy: patients were subjected to general anesthesia via an orotracheal tube. Then the child was placed in supine in the Rose position and Boyle Davis mouth gag was inserted as in tonsillectomy. Then St. Clair Thompson adenoid curette was used to perform a conventional adenoidectomy. A 70° endoscope was used for assessment of the presence of posterior pharyngeal wall injury or remnant of adenoid tissue. Ribbon gauze was placed in the nasopharynx for attaining hemostasis and was removed after 5 min.

Endoscopic microdebrider-assisted adenoidectomy: patients were subjected to General Anesthesia via an orotracheal tube. Then the child was placed in supine in the Rose position and Boyle Davis mouth gag was inserted as in tonsillectomy. With the use of endoscope, the Microdebrider unit was set at 3000 rpm oscillating mode to remove the adenoid tissue from the choanal and tubaric regions transorally. Once all the adenoid tissue was removed, ribbon gauze was placed in the nasopharynx for attaining hemostasis and was removed after 5 min. Microdebrider with special adenoid blade, which is longer, has a window on convex side for use transorally to adapt to the roof of nasopharynx. A 70° sinoscope and angled microdebrider was passed transorally. The adenoidectomy was started high in the nasopharynx from upper limit of adenoid tissue, which often cannot be reached by conventional curette. Resection was continued from one side to another side fashion on an even level until the inferior edge of adenoid pad will be reached. The cutting and aspirating action of the shaver and simultaneous irrigation removes both adenoid tissue and the blood, thus providing a clear view. Postoperative care was given and patient was discharged the same day.

**Fig. 1:**
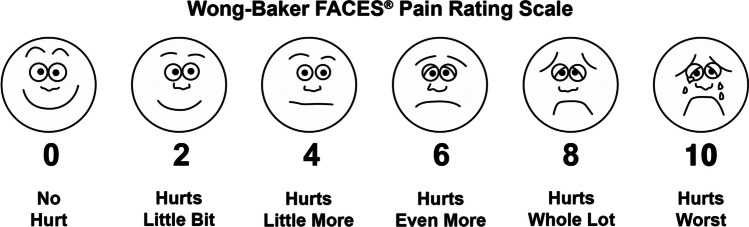
Pain scale according to facial expression [8]

Postoperative endoscopic assessment of any adenoid remnant after 6 months was done. Postoperative bleeding was evaluated postoperatively. The patients’ postoperative pain assessment were compared using VAS from 0 to 5 before operation, after 2 weeks, and 3 months after adenoidectomy as shown in Fig. 1.

Data were fed to the computer and analyzed using IBM SPSS software package version 20.0***.* (**Armonk, NY: IBM Corp**)**. Qualitative data were described using number and percent. The Shapiro–Wilk test was used to verify the normality of distribution. Quantitative data were described using range (minimum and maximum), mean, standard deviation, median and interquartile range (IQR). Significance of the obtained results was judged at the 5% level. The used tests were Chi-square test for categorical variables, to compare between different group, Monte Carlo correction for correction for chi-square when more than 20% of the cells have expected count less than 5, Student *t*-test for normally distributed quantitative variables, to compare between two studied groups, and *F*-test (ANOVA) for normally distributed quantitative variables, to compare between more than two groups.

## Results

A total of 90 patients were included in our prospective study. The mean age in group I was 9.03 years and in group II, it was 8.99 years and was 8.99 years in group III with insignificant differences between three groups and in regard to sex in group I, 60% were males and 40% were females, in group II: 50% were males and 50% were females and in group III: 50% were male and 50% were female displaying insignificant differences between the three groups (Table 1).

**Table 1:** Demographic data

	Group I (*n* = 30)		Group II (*n* = 30)		Group III (*n* = 30)		Test of Sig	*p*
	No	%	No	%	No	%		
Sex								
Male	18	60.0	15	50.0	15	50.0	*χ*^2^ = 0.804	0.669
Female	12	40.0	15	50.0	15	50.0		
Age (years)								
Min.–Max	5.80 – 12.0		5.70–12.0		5.70–12.0		*F* = 0.004	0.996
Mean ± SD	9.03 ± 1.87		8.99 ± 2.08		8.99 ± 2.08			
Median (IQR)	9.35 (7.40 – 10.20)		8.90 (7.40–10.90)		8.90 (7.40–10.90)			

*IQR* inter quartile range, *SD* standard deviation, *χ*^*2*^ Chi square test, *F* *F* for One way ANOVA test

*p* *p* value for comparing between the three studied groups

In regard to adenoid tissue, using endoscopic grading, there is significant differences between preoperative and postoperative grading for each group separately. There are non-significant differences comparing the three groups as preoperative and postoperative grading (Table 2).

**Table 2:** Pre- and post-operative endoscopic grading of adenoid tissue

Endoscopic assessment	Group I (*n* = 30)		Group II (*n* = 30)		Group III (*n* = 30)		χ^2^	*p*
	No	%	No	%	No	%		
Preoperative assessment								
Grade 1	1	3.33	2	6.66	1	3.33	0.892	0.812
Grade 2	8	26.67	8	26.67	6	20.0		
Grade 3	10	33.33	12	40.0	13	43.34		
Grade 4	11	36.67	8	26.67	10	33.33		
Postoperative assessment								
Grade 1	21	70.0	25	50.0	26	50.0	2.918	0.134
Grade 2	4	30.0	3	50.0	3	50.0		
Grade 3	3	30.0	2	50.0	1	50.0		
Grade 4	2	10.0	0	10.0	0	10.0		
*χ*^2^	6.412		8.915		9.451			
*p*	0.45		0.012		0.008			

*χ*^*2*^ Chi square test, *p* *p* value

As regard grading of adenoid hypertrophy preoperative was in group I 3.33% grade 1, 26.67% grade II, 33.33% grade III, 36.67% grade IV, in group II 6.66% grade I, 26.67% grade II, 40% grade III, 26.67% grade IV, in group III 3.33% grade I, 20% grade II, 43.34% grade III and 33.33% grade IV with insignificant between three groups (Table 2).

Preoperatively, all cases were type A tympanogram but postoperative in group I after 2 weeks 91.7% had A, 3.3% had AS, and 5% had C; after 3 months, 95% had A and 5% had C; after 6 months, all were A, in group II 80/5 had A, 3.3% had AS, 8.3% had C, 1.7% had B1, 6.7% had B2 after 2 weeks, but after 3 months 85% had A, 8.3% had C, 6.7% had B2, and after 6 months 85% had A, 6.7% had C, 1.7% had B1, and 6.7% had B2. In group III, after 2 weeks, 90% had A, 3.3% had AS, 5% had C, and 1.7% had B2; after 3 months, 93.3% had A, 5% had C, 1.7% had B2 and after 6 months 95% had A and 5% had C as shown in Table 3.

**Table 3:** Comparison between the three studied groups according to tympanogram for each ear

Tympanogram	Group I (*n* = 60)		Group II (*n* = 60)		Group III (*n* = 60)		*χ*^2^	^MC^*p*
	No	%	No	%	No	%		
Pre-operative								
A	60	100.0	60	100.0	60	100.0	–	–
B	0	0	0	0.0	0	0.0		
Post-operative								
After 2 weeks								
A	55	91.7	48	80.0	54	90.0	7.488	0.453
AS	2	3.3	2	3.3	2	3.3		
C	3	5.0	5	8.3	3	5.0		
B1	0	0.0	1	1.7	0	0.0		
B2	0	0.0	4	6.7	1	1.7		
After 3 months								
A	57	95.0	51	85.0	56	93.3	5.279	0.228
AS	0	0.0	0	0.0	0	0.0		
C	3	5.0	5	8.3	3	5.0		
B1	0	0.0	0	0.0	0	0.0		
B2	0	0.0	4	6.7	1	1.7		
After 6 months								
A	60	100.0	51	85.0	57	95.0	12.203^*^	0.008^*^
AS	0	0.0	0	0.0	0	0.0		
C	0	0.0	4	6.7	3	5.0		
B1	0	0.0	1	1.7	0	0.0		
B2	0	0.0	4	6.7	0	0.0		

*χ*^*2*^ Chi square test, *MC*: Monte Carlo, *p* *p* value for comparing between the three studied groups.

*Statistically significant at *p* ≤ 0.05

Postoperative bleeding occurs in 20% of cases in group I only with significant differences between three groups as shown in Table 4.

**Table 4:** Post-operative bleeding

Post-operative bleeding	Group I (*n* = 30)		Group II (*n* = 30)		Group III (*n* = 30)		*χ*^2^	^MC^*p*
	No	%	No	%	No	%		
No	24	80.0	30	100.0	30	100.0	10.085^*^	0.004^*^
Yes	6	20.0	0	0.0	0	0.0		

*χ*^*2*^ Chi square test, *MC*: Monte Carlo, *p* *p* value for comparing between the three studied groups.

*Statistically significant at *p* ≤ 0.05

Mean VAS in day 1 was 1.73 in group I, 1.13 in group II, in group III it was 0.17 and changed to 0.5, 0.33, and 0.03 respectively in day 7. There was a significant decrease in VAS in cases in group III after 1, 7 days than other groups as Table 5.

**Table 5:** Visual analogue score for pain assessment

Pain VAS score	Group I (*n* = 30)	Group II (*n* = 30)	Group III (*n* = 30)	*H*	*p*
Day 1					
Min.–Max	0.0–3.0	0.0–2.0	0.0–2.0	41.079^*^	< 0.001^*^
Mean ± SD	1.73 ± 0.87	1.13 ± 0.82	0.17 ± 0.46		
Median (IQR)	2.0(1.0–2.0)	1.0(0.0–2.0)	0.0(0.0–0.0)		
Sig. bet. groups	*p*_1_ = 0.031^*^, *p*_2_ < 0.001^*^, *p*_3_ < 0.001^*^				
Day 7					
Min. – Max	0.0–2.0	0.0–2.0	0.0–1.0	11.756^*^	0.003^*^
Mean ± SD	0.50 ± 0.68	0.33 ± 0.55	0.03 ± 0.18		
Median (IQR)	0.0 (0.0–1.0)	0.0 (0.0–1.0)	0.0 (0.0–0.0)		
Sig. bet. Groups	*p*_1_ = 0.319, *p*_2_ = 0.001^*^, *p*_3_ = 0.019^*^				

*χ*^*2*^ Chi square test, *p* *p* value for comparing between the three studied groups

*Statistically significant at *p* ≤ 0.05

## Discussion

Adenoid enlargement is an important cause for Eustachian tube dysfunction and recurrent otitis media in pediatric population. In recent study by Somayaji et al., incidence of adenoid hypertrophy was more in 4–6-year age group, followed by 7–9-year age group. This observation was similar to the study by Fujioka et al., which showed that the size of adenoid varies among children, but the maximum size was between 4 and 8 years age, which then regress gradually by 15 years of age. There was no significant difference in the gender distribution of incidence of adenoid hypertrophy [9, 10].

Children with OME may remain asymptomatic. Some present with deafness or ear fullness. In the present study, 12 children (22.2%) had hearing impairment. This incidence varies depending on the study. In a similar study by Kindermann et al. to investigate whether the obstruction of the Eustachian tube orifice due to adenoid hyperplasia causes a change in the MEP showed that the incidence of hearing loss to be 16% (8 out of 50 children). Sarafoleanu et al., in their study, showed the implications of adenoid tissue hypertrophy in the genesis of Eustachian tube dysfunction observed the incidence of hypoacusis to be 77.8%. In another study by Khayat and Dabbagh to identify the incidence of OME in children with adenoid hypertrophy, 22 (50%) out of 44 children with OME had deafness which was detected by statements of parents and teachers. Enache et al. in his study on 119 children with OME observed that all children presented with a history of deafness [11, 12].

In a study by Somayaji et al., otoscopy findings showed varied appearance of TM. 24% (26 ears) showed air–fluid level and 17.6% (19 ears) had dull and retracted TM. Tympanogram results revealed pathological curves in 45 ears (25% Type B curve and 16.7% Type C curve). Sarafoleanu et al. in his study observed that 28.57% had retracted TM and 49.2% had middle ear effusion. Furthermore, tympanogram results showed pathologic curves in 98 cases (40.47% Type B curve and 37.30% Type C curve). In a similar study by Khayat and Dabbagh also, the otoscopic findings were varied with distorted cone of light, retraction of TM, and air bubbles. A study by Ajayan et al. conducted to study the effect of adenoidectomy with tonsillectomy in pediatric patients with OME, showed dull and retracted TM in 78.5% cases and air–fluid was seen only in 11.42% [9, 11].

In a study by Somayaji et al., the difference between the two techniques (one group for conventional adenoidectomy and another for microdebrider adenoidectomy) was statistically significant (*P* < 0.001). Furthermore, 69.1% and 17.2% with Type A tympanogram changed to Type C and Type B, respectively, in the immediate postoperative period [9].

These observations were similar to the study by Choi et al. to evaluate the effect of adenotonsillectomy on immediate Eustachian tube function in children with adenotonsillar hypertrophy. He observed that majority had C Type of curve. On postoperative day 2, 76% (38/50) of cases were unresolved (CC and BB types), while 24% (12/50) were normal (AA type) or resolved (CA type). There was a statistically significant difference (*P* < 0.001) in bilateral MEPs between preoperative and postoperative days [13].

Similar results were observed in a study by Unlu et al. to analyze the changes in the MEP in the early period after adenoidectomy in children with adenoid hypertrophy without OME. He observed that there was a pathological decrease in the MEP 24 h after the procedure in one ear in 48 patients (75%) and bilateral Eustachian dysfunction in 38 patients (59.3%). Furthermore, Type B tympanogram was not seen in any patients, postoperatively. These changes in MEP returned to preoperative value by 7th postoperative day [6].

The increase in hearing threshold and reduction in MEP in the immediate period could be due to temporary dysfunction of Eustachian tube due to edema or blood clots at the surgical site, especially around Eustachian tube orifice. Moreover, due to pain in the postoperative period, swallowing is reduced, and this also adds on to the cause for Eustachian tube dysfunction [9].

In a study by Somayaji et al., there was a significant improvement in the MEP and hearing threshold at 6 weeks following surgery as seen with PTA and tympanometry. There was a complete resolution of OME in 15 out of 16 children as suggested by postoperative Type A tympanogram. Similar results were observed in a study by Tuohimaa and Palva. A similar study by Zaman and Borah also showed that there was a significant improvement in the MEP following adenoidectomy [9, 14, 15].

Sandooja et al. in his study on effect of adenotonsillectomy on hearing threshold and MEP also has shown improvement in MEP and hearing threshold following surgery and hence concluded that adenoidectomy improves the Eustachian tube function and MEP by eliminating the mechanical obstruction, edema of Eustachian tube orifice due to repeated infection [16].

Similar results were observed in a retrospective study by Enache et al. At 1 month after surgery, a normal Eustachian tube function was seen in 43.70% of the children and at 3 months after surgery, when the second reassessment was made, 109 children (91.60%) presented a total recovery of the middle ear function with a normal Eustachian tube activity [17].

Ajayan et al., in his study, observed that 6 weeks following surgery, 55.71% of Type B tympanogram changed to Type A and 15.7% to Type C, while in 28.57%, Type B persisted. After 3 months, 65.71% of tympanogram were Type A and 8.57% were Type C. In 25.71% of subjects, Type B tympanogram persisted. It was also observed that, in all those subjects with persistent Type B tympanogram, preoperative hearing loss was higher, that is, between 40 and 50 dB [18].

In the present study, one case (2%) had persistent OME bilaterally as suggested by persistence of Type B tympanogram and reduced MEP. This could be due to persistence of Eustachian tube dysfunction [9].

In another study, comparing effect of tonsillectomy and adenotonsillectomy over the tubal function, they discussed that there is a transient negative middle ear pressure in the early postoperative period more after adenotonsillectomy, then adenoidectomy and least after tonsillectomy with complete resolution one month postoperatively [19].

Cost difference between the coblation and microdebrider blade versus conventional technique is always matter of discussion in different techniques of adenoidectomy. Though cost assessment was not studied as primary outcome of the present study, coblation technique had cost more than for microdebrider or conventional techniques. Although, coblation had better results on postoperative tympanographic findings but it had more cost with less bleeding rather than conventional technique. Microdebrider technique had less cost with mostly similar results in tympanographic findings and bleeding outcomes rather than coblation technique.

## Conclusion

There are better results in tympanographic data at conventional adenoidectomy versus other techniques. However, there are also better postoperative results after either coblation or endoscopic microdebrider adenoidectomy over the conventional technique.

## Data Availability

The data sets used and/or analyzed in the current study are available from the corresponding author on reasonable request.
